# Amiodarone and the thyroid dysfunction

**DOI:** 10.1186/1756-6614-6-S2-A23

**Published:** 2013-04-05

**Authors:** Helena Jastrzębska

**Affiliations:** 1 Endocrinology Department of Medical Centre of Postgraduate Education, Warsaw, Poland

## 

Amiodarone, a class III antiarrhytmic agent, is a benzofuran derivative containing 75 mg iodide per 200 mg tablet. During the metabolism of 200 mg of the drug approximately 6-8 mg of inorganic iodine is released into the systemic circulation. Amiodarone is very lipophilic and concentrates in various tissues and organs such as adipose tissue, skeletal muscles, myocardium, liver, lung and thyroid. Amiodarone is dealkylated in the liver to its major active metabolite desethylamiodarone. Amiodarone therapy is associated with a number of side effects, including thyroid dysfunction – thyrotoxicosis in 2-15% and hypothyroidism in 5-20% of patients, respectively. The effects of amiodarone on thyroid function depend on underlying thyroid status and dietary iodine intake. Patients with autoimmune thyroid disease are more likely to develop hypothyroidism due to failure to escape from Wolff-Chaikoff effect. In patients with multinodular goiter or latent Graves’ disease hyperthyroidism may occur. Amiodarone may also cause destructive thyroiditis in patients without underlying thyroid disease. Thyrotoxicosis may be a result of increased synthesis of thyroid hormones (type 1) or of their excessive release due to a direct damage of thyroid cells caused by amiodarone, its metabolite desethylamiodarone or iodine (type 2). The distinction between type 1 and type 2 thyrotoxicosis is crucial, since therapy is different in these two types. The differential diagnosis is based on the presence of goiter, evaluation of thyroid autoantibodies, colour flow Doppler image, thyroid uptake and on the response to steroids or perchlorate. Patients with type 1 thyrotoxicosis require thionamides or potassium/sodium perchlorate, while those with type 2 – corticosteroids. Hypothyroidism is treated with L-thyroxine. The signs and symptoms of amiodarone-induced thyrotoxicosis and hypothyroidism can be scanty. Therefore, all patients treated with amiodarone need periodic examination of thyroid function. Apart from inducing thyroid dysfunction, amiodarone causes hypercholesterolemia due to decreased expression of the LDL receptor gene, which is regulated by T3. Adverse effects of amiodarone have led to the search for analogues with the same efficacy but safer profile. Dronedarone is structurally related to amiodarone but does not contain iodine atoms and does not increase the incidence of thyroid disease. Dronedarone appears to be less-effective but may be beneficial for patients with atrial fibrillation or flutter who are at risk of developing amiodarone induced thyroid dysfunction.

